# Prevalence and clinical correlates of suicide attempts in patients with first-episode drug-naïve major depressive disorder and comorbid autoimmune thyroiditis

**DOI:** 10.1192/bjo.2024.48

**Published:** 2024-04-30

**Authors:** Yinli Luo, Yanan Zhou, Pu Peng, Ning Yuan, Xiangyang Zhang

**Affiliations:** Department of Psychiatry, Hunan Brain Hospital, Hunan Second People's Hospital, Changsha, China; Department of Psychiatry and National Clinical Research Center for Mental Disorders, The Second Xiangya Hospital of Central South University, China; CAS Key Laboratory of Mental Health, Institute of Psychology, Chinese Academy of Sciences, Beijing, China; and Department of Psychology, University of Chinese Academy of Sciences, Beijing, China

**Keywords:** Anxiety, autoimmune thyroiditis, major depressive disorder, suicide attempts, first-episode drug-naïve

## Abstract

**Background:**

Autoimmune thyroiditis is closely associated with major depressive disorder (MDD) and suicide attempts. However, few studies have examined this relationship.

**Aims:**

The study aimed to assess the prevalence and correlates of suicide attempts in patients with first-episode drug-naïve (FEDN) MDD and autoimmune thyroiditis.

**Method:**

We recruited 1718 out-patients with FEDN MDD and assessed depressive, anxiety and psychotic symptoms with the Hamilton Rating Scale for Depression, Hamilton Rating Scale for Anxiety (HRSA) and Positive and Negative Syndrome Subscale positive subscale, respectively. The serum levels of free triiodothyronine, free thyroxine, thyroid stimulating hormone (TSH), antithyroglobulin, thyroid peroxidase antibody (TPOAb) and several other metabolic parameters were assessed. Patients were divided into non-autoimmune thyroiditis, autoimmune thyroiditis only and autoimmune thyroiditis with abnormal TSH groups, based on autoimmune thyroiditis severity. Multiple logistic regression model was applied to identify the correlates of suicide attempts in patients with MDD and autoimmune thyroiditis with abnormal TSH.

**Results:**

Compared with the non-autoimmune thyroiditis group, the autoimmune thyroiditis with abnormal TSH group had a nearly fourfold higher likelihood of reporting a suicide attempt, whereas no difference was found between the non-autoimmune thyroiditis and autoimmune thyroiditis only groups. HRSA score, lnTPOAb and lnTSH were independently associated with suicide attempts in patients with autoimmune thyroiditis with abnormal TSH.

**Conclusions:**

Patients with MDD and autoimmune thyroiditis with abnormal TSH are at higher risk for suicide attempt. TPOAb, TSH and anxiety are all independently associated with suicide attempts in this population, and regular thyroid checks are warranted.

Major depressive disorder (MDD) is one of the most common and debilitating psychiatric disorders, affecting 4.4% of the general population worldwide.^1^ Suicide is the most serious complication of MDD. A recent meta-analysis showed that patients with MDD are 7.34 times more likely to have a suicide attempt in the past year than patients without this disorder.^2^ In China, the estimated prevalence of suicide attempt in patients with MDD is 23.7%.^3^ The identification of biomarkers for suicide attempt was essential for early detection and intervention among these patients.

The dysfunction of the hypothalamic-pituitary-thyroid (HPT) axis might play a role in the pathophysiology of MDD and suicide attempt. The link between thyroid dysfunction and depression has been well established. One recent meta-analysis of 348 014 participants demonstrated a positive relationship between clinical depression and hypothyroidism.^4^ Thyroid dysfunction, such as blunted thyroid stimulating hormone (TSH) response to thyrotropin-releasing hormone, was common in MDD. Furthermore, thyroid dysfunction in MDD is closely linked to various clinical outcomes. These include metabolic disturbances,^5^ an elevated risk of readmission^6^ and more severe psychiatric symptoms.^7^ The effectiveness of thyroid hormone therapy in treating depression, supported by several studies,^7,8^ further highlights the critical role of thyroid function in the context of MDD.

More importantly, emerging studies have indicated a link between thyroid dysfunction, particularly increased TSH levels, and suicide attempt. For example, Fu et al conducted a meta-analysis on the association between blood hormones and suicidal behavior.^9^ They found significantly higher TSH levels in patients with a history of suicide attempt compared with those without.^9^ Recent studies in patients with MDD have produced similar results, finding higher TSH levels could be potential biomarkers of suicide attempt.^10–13^

## Association between autoimmune thyroiditis, suicide attempts and major depressive disorder

Autoimmune thyroiditis is the most common autoimmune disease in humans, which is characterised by abnormal autoimmune thyroid antibodies. Prior studies have established a positive link between autoimmune thyroiditis and increased rates of depression and suicide attempt in the general population.^14,15^ However, the relationship between suicide attempt and autoimmune thyroiditis in patients with MDD remains unclear. Recent research on 1279 out-patients and 1589 in-patients with MDD indicated higher autoimmune thyroid antibody levels in those with a history of suicide attempt,^10,12^ suggesting a potential role of autoimmune thyroiditis in MDD-related suicide attempt. Yet, these studies did not further investigate suicide attempt predictors in patients with MDD with autoimmune thyroiditis. A more detailed investigation into the associated factors for suicide attempt in MDD with autoimmune thyroiditis could pave the way for early identification and targeted intervention strategies in this patient group.

## The present study

Therefore, the present study aimed to (a) investigate the independent association between autoimmune thyroiditis and suicide attempt in patients with first-episode drug-naïve (FEDN) MDD, thereby minimising the impact of illness duration and medication; and (b) determine the prevalence and clinical correlates of suicide attempt in patients with MDD and comorbid autoimmune thyroiditis.

## Method

### Participants and procedure

This study was conducted in the Outpatient Department of Psychiatry of the First Hospital of Shanxi Medical University, China, from 2015 to 2017. Patients who met the following criteria were eligible for inclusion: (a) diagnosed with MDD by two psychiatrists, according to DSM-IV criteria; (b) scored >23 on the 17-item Hamilton Rating Scale for Depression (HRSD), (c) aged 18–60 years and Han Chinese; (d) in their first MDD episode at the time of enrolment, with the duration of this episode not exceeding 24 months; and (e) have not received any medications, such as antidepressants, antipsychotics or thyroxine treatment. Patients meeting any of the following criteria were excluded: (a) being pregnant or having a haemorrhage, (b) comorbid with another serious DSM-IV axis I disorder or serious medical conditions, (c) with any substance use disorders other than tobacco use and (d) refusal to provide written informed consent.

The authors assert that all procedures contributing to this work comply with the ethical standards of the relevant national and institutional committees on human experimentation and with the Helsinki Declaration of 1975, as revised in 2008. This study was approved by the Institutional Review Board of the First Hospital of Shanxi Medical University (approval number 2016-Y27). All of the participants provided written informed consent.

### Clinical interview and assessment

The participants’ basic information was collected with a self-administered questionnaire that covered age, gender, level of education, marital status, duration of MDD and time of onset.

All patients were interviewed by two trained psychiatrists independently, based on the Structured Clinical Interview for DSM-IV, and MDD was diagnosed according to DSM-IV criteria. For each patient, depression, anxiety and psychotic symptoms were assessed with the HRSD, Hamilton Rating Scale for Anxiety (HRSA) and positive scale of the Positive and Negative Syndrome Scale (PANSS), respectively. The three scales are highly validated and widely used in the MDD population.^16^ Cut-off points of 21 for the HRSA and 15 for the PANSS were used to screen for anxious depression and psychotic depression.^17^ The interrater correlation coefficients of all three scales were above 0.8.

Suicide attempts were assessed via face-to-face interviews. All participants were asked to answer the question ‘Have you ever attempted suicide in your lifetime?’, and those who responded with ‘yes’ were recorded as ever having a suicide attempt. These participants were further inquired about the exact date, frequency and method of their suicide attempts, and their families were contacted for more details about the suicide attempts when necessary.

### Biochemical indexes

Fasting blood samples were collected from all of the participants before any treatment was administered, and serum free triiodothyronine (FT3), free thyroxine (FT4), TSH, antithyroglobulin (TgAb), thyroid peroxidase antibody (TPOAb), total cholesterol, total triglycerides, high-density lipoprotein (HDL-C), low-density lipoprotein (LDL-C) and glucose were measured. Patients’ weight and height were also measured, and body mass index (BMI) was calculated with the formula: BMI = weight (kg)/height (m^2^). Based on prior studies on Chinese populations,^18^ the normal range is 0–34 IU/ml for TPOAb and 0–115 IU/ml for TgAb. Patients with serum levels of TPOAb and/or TgAb higher than the reference values were considered positive for autoimmune thyroiditis. We further divided the autoimmune thyroiditis patients into autoimmune thyroiditis only (those with elevated autoimmune thyroid antibodies and normal TSH levels) and autoimmune thyroiditis with abnormal TSH (those with elevated autoimmune thyroid antibodies and TSH levels exceeding 4.2 mIU/L) groups, to reflect the severity of autoimmune thyroiditis.

### Statistical analysis

First, we performed log-transformation of metabolic indexes, including TSH, FT3, FT4, TPOAb, TgAb, HDL-C, LDL-C, total cholesterol, total triglycerides and glucose. The log-transformed parameters were used for comparison, correlation and regression analysis, whereas the original data was employed for description. Continuous variables were presented as mean (s.d.), whereas categorical variables were presented as percentages and frequencies. The intergroup comparison was performed with the chi-squared test, Student *t*-tests and analysis of variance, as appropriate. Pearson correlation analysis was used to determine the association between thyroid indexes and clinical symptoms.

Second, we performed the least absolute shrinkage and selection operator (LASSO) regression to determine whether autoimmune thyroiditis severity could be an independent associated factor for suicide attempt. The initial model incorporated variables across various domains: autoimmune thyroiditis severity (categorised as non-autoimmune thyroiditis, autoimmune thyroiditis only, and autoimmune thyroiditis with abnormal TSH), demographic information (including age, age at onset, duration of illness, gender, marital status and education level), clinical symptoms (HRSD scores, HRSA scores and PANSS scores) and metabolic parameters (log-transformed values of TSH (lnTSH), thyroid peroxidase antibody (lnTPOAb), antithyroglobulin (lnTgAb), FT3, FT4, total cholesterol, total triglycerides, HDL-C, LDL-C, glucose, systolic blood pressure, diastolic blood pressure and BMI). The optimal LASSO model was selected based on a ten-fold cross-validation approach within 1 s.d. of the minimum. Subsequently, the variables identified by the LASSO regression were incorporated into the multivariate logistic regression model for suicide attempt in all patients with MDD, using a stepwise method.

Finally, we further evaluated the associated factors of suicide attempt in patients with autoimmune thyroiditis. Both univariate and multivariate analyses were performed. Significant variables in the univariate analysis (*P* < 0.05) were included in the multivariate regression model for suicide attempt, by a stepwise method.

## Result

### Characteristics of the participants

A total of 1718 patients with FEDN MDD (588 men and 1130 women) were recruited. The mean age was 35 years, and the mean duration of MDD was 6.3 months. According to the HRSA and PANSS cut-off values, the prevalence of anxious depression and psychotic depression was 52% (*n* = 894) and 10% (*n* = 171), respectively.

Patients were categorised into three groups: non-autoimmune thyroiditis (*n* = 1214), autoimmune thyroiditis only (*n* = 105) and autoimmune thyroiditis with abnormal TSH (*n* = 399), based on serum TSH and autoimmune thyroid antibody levels. Significant differences were observed among these groups in demographic data, clinical symptoms and metabolic indices (Table 1). The autoimmune thyroiditis with abnormal TSH group experienced longer illness duration (7.3 months) compared with the non-autoimmune thyroiditis (6.1 months) and autoimmune thyroiditis only (5.3 months) groups (*P* < 0.001). This group also showed more pronounced clinical symptoms and metabolic disturbances than the other two groups. They had notably higher blood pressure, total cholesterol, total triglycerides, LDL-C and glucose levels, along with lower HDL-C levels, compared with both the non-autoimmune thyroiditis and autoimmune thyroiditis only groups. Interestingly, the severity of anxiety and psychotic symptoms did not differ between the non-autoimmune thyroiditis and autoimmune thyroiditis only groups, but the autoimmune thyroiditis only group exhibited less severe depressive symptoms than the non-autoimmune thyroiditis group.

**Table 1:** Sample characteristics of patients with major depressive disorder based on autoimmune thyroiditis severity

Characteristic	Autoimmune thyroiditis severity				*P*-value^b^
	Overall, *N* = 1 718^a^	Non-autoimmune thyroiditis, *n* = 1 214^a^	Autoimmune thyroiditis only, *n* = 105^a^	Autoimmune thyroiditis with abnormal TSH, *n* = 399^a^	
Age, years	35 (12)	34 (12)	35 (12)	36 (13)	0.024
Duration, months	6.3 (4.7)	6.1 (4.6)	5.4 (4.5)	7.3 (5.1)	<0.001
Age at onset, years	35 (12)	34 (12)	34 (12)	36 (12)	0.030
Gender					0.37
Male	588 (34%)	427 (35%)	36 (34%)	125 (31%)	
Female	1130 (66%)	787 (65%)	69 (66%)	274 (69%)	
Education level					0.52
Below college	1173 (68%)	825 (68%)	77 (73%)	271 (68%)	
College or above	545 (32%)	389 (32%)	28 (27%)	128 (32%)	
Married					0.17
Unmarried	502 (29%)	370 (30%)	25 (24%)	107 (27%)	
Married	1216 (71%)	844 (70%)	80 (76%)	292 (73%)	
HRSD scores	30.30 (2.94)	30.02 (2.89)	28.86 (3.04)	31.50 (2.70)	<0.001
HRSA scores	20.8 (3.5)	20.4 (3.3)	20.6 (3.5)	22.1 (3.6)	<0.001
Anxious depression	894 (52%)	579 (48%)	54 (51%)	261 (65%)	<0.001
PANSS scores	8.9 (4.4)	8.6 (4.1)	8.1 (3.3)	9.9 (5.3)	<0.001
Psychotic depression	171 (10.0%)	101 (8.3%)	7 (6.7%)	63 (16%)	<0.001
Suicide attempts	346 (20%)	176 (14%)	18 (17%)	152 (38%)	<0.001
TSH, uIU/L	5.07 (2.56)	4.55 (2.33)	2.72 (1.19)	7.26 (2.08)	
TgAb, IU/L	90 (238)	21 (13)	225 (314)	266 (411)	
TPOAb, IU/L	72 (164)	16 (7)	159 (223)	221 (262)	
FT3, pmol/L	4.90 (0.72)	4.90 (0.71)	4.91 (0.80)	4.93 (0.74)	
FT4, pmol/L	16.7 (3.1)	16.7 (3.1)	16.1 (3.2)	16.7 (3.0)	
Glucose, mmol/L	5.40 (0.65)	5.35 (0.63)	5.11 (0.54)	5.62 (0.66)	
Total cholesterol, mmol/L	5.25 (1.11)	5.13 (1.03)	4.64 (0.95)	5.78 (1.17)	
HDL-C, mmol/L	1.22 (0.29)	1.25 (0.27)	1.30 (0.26)	1.11 (0.31)	
LDL-C, mmol/L	2.98 (0.86)	2.92 (0.86)	2.64 (0.75)	3.27 (0.83)	
Total triglycerides, mmol/L	2.17 (0.99)	2.14 (0.98)	2.02 (0.97)	2.29 (1.01)	
BMI, kg/m^2^	24.37 (1.92)	24.39 (1.90)	23.80 (1.64)	24.44 (2.04)	0.007
SBP, mmHg	119 (11)	118 (11)	115 (11)	125 (9)	<0.001
DBP, mmHg	76 (7)	75 (7)	74 (6)	79 (7)	<0.001
LnTSH	1.45 (0.65)	1.35 (0.65)	0.85 (0.64)	1.94 (0.28)	<0.001
LnFT3	1.58 (0.15)	1.58 (0.15)	1.58 (0.17)	1.58 (0.15)	0.76
LnFT4	2.80 (0.19)	2.80 (0.19)	2.76 (0.21)	2.80 (0.18)	0.11
LnTPOAb	3.26 (1.18)	2.67 (0.44)	4.41 (1.14)	4.75 (1.21)	<0.001
LnTgAb	3.46 (1.16)	2.91 (0.45)	4.65 (1.30)	4.83 (1.26)	<0.001
LnGlucose	1.68 (0.12)	1.67 (0.12)	1.63 (0.10)	1.72 (0.12)	<0.001
LnTC	1.64 (0.22)	1.61 (0.21)	1.51 (0.20)	1.73 (0.21)	<0.001
LnTG	0.67 (0.45)	0.66 (0.46)	0.60 (0.47)	0.74 (0.43)	0.003
LnLDL-C	1.05 (0.31)	1.03 (0.31)	0.93 (0.29)	1.15 (0.28)	<0.001
LnHDL-C	0.17 (0.25)	0.20 (0.23)	0.24 (0.21)	0.07 (0.29)	<0.001

TSH, thyroid stimulating hormone; HRSD, Hamilton Rating Scale for Depression; HRSA, Hamilton Rating Scale for Anxiety; PANSS, Positive and Negative Syndrome Scale; TPOAb, thyroid peroxidase antibody; TgAb, antithyroglobulin; FT3, free triiodothyronine; FT4, free thyroxine; HDL-C, high-density lipoprotein; LDL-C, low-density lipoprotein; BMI, body mass index; SBP, systolic blood pressure; DBP, diastolic blood pressure; LnTSH, log-transformed TSH; LnFT3, log-transformed FT3; LnFT4, log-transformed FT4; LnTPOAb, log-transformed thyroid peroxidase antibody; LnTgAb, log-transformed antithyroglobulin; LnGlucose, log-transformed glucose; LnTC, log-transformed total cholesterol; LnTG, log-transformed total triglycerides; LnLDL-C, log-transformed LDL-C; LnHDL-C, log-transformed HDL-C.

a. Mean (s.d.) or *n* (%).

b. One-way analysis of variance or Pearson's chi-squared test.

The association of thyroid indices with clinical symptoms was evaluated with Pearson correlation, as detailed in Supplementary Table 1 available at https://doi.org/10.1192/bjo.2024.48. Notably, lnTSH showed a positive association with HRSD scores (*r* = 0.429, *P* < 0.001), HRSA scores (*r* = 0.234, *P* < 0.001) and PANSS scores (*r* = 0.276, *P* < 0.001). Additionally, lnTPOAb and lnTgAb were significantly correlated with clinical symptoms (*r* = 0.132–0.211, *P* < 0.001).

### Association between suicide attempt and autoimmune thyroiditis

Among the FEDN MDD cohort, 346 (20%) patients reported having made a lifetime suicide attempt, with occurrences distributed as follows: 254 (73%) once, 65 (19%) twice and 27 (8%) three or more times. The analysis of 455 total suicide attempts revealed medication overdose (131 cases) as the most common method, followed by jumping (86 cases), cutting (83 cases), vehicular impact (53 cases) and gas poisoning (32 cases).

The prevalence of suicide attempt was 14% (*n* = 176) in the non-autoimmune thyroiditis group, 17% (*n* = 18) in the autoimmune thyroiditis only group and 38% (*n* = 152) in autoimmune thyroiditis with abnormal TSH group. Univariate logistic regression analysis revealed that, compared with the non-autoimmune thyroiditis group, the autoimmune thyroiditis with abnormal TSH group had a nearly fourfold higher likelihood of reporting suicide attempt (odds ratio 3.63, 95% CI 2.80–4.70; *P* < 0.001). No significant difference in suicide attempt prevalence was observed between the autoimmune thyroiditis only and non-autoimmune thyroiditis groups (Table 2). Five variables were selected as best predictors for suicide attempt in patients with FEDN MDD, including autoimmune thyroiditis severity, lnTSH, HRSA scores, HRSD scores and systolic blood pressure, using the LASSO regression method (Supplementary Fig. 1). We conducted a multivariate logistic regression model using these five predictors. The results indicate that patients with autoimmune thyroiditis and abnormal TSH levels were twice as likely to report a history of suicide attempt compared with the non-autoimmune thyroiditis group (odds ratio 2.26, 95% CI 1.69–3.02; *P* < 0.001). In contrast, no significant difference in the prevalence of suicide attempt was observed between the autoimmune thyroiditis only group and the non-autoimmune thyroiditis group.

**Table 2:** Association of autoimmune thyroiditis severity with suicide attempt

Autoimmune thyroiditis severity	Without suicide attempt	With suicide attempt,	Crude odds ratio (95% CI)	*P*-value	Adjusted odds ratio (95% CI)^a^	*P*-value
Non-autoimmune thyroiditis	1038 (86%)	176 (14%)	Reference	Reference	Reference	Reference
Autoimmune thyroiditis only	87 (83%)	18 (17%)	1.22 (0.72–2.08)	0.463	1.46 (0.81–2.65)	0.212
Autoimmune thyroiditis with abnormal TSH	247 (62%)	152 (38%)	3.63 (2.80–4.70)	<0.001	2.26 (1.69–3.02)	<0.001

TSH, thyroid stimulating hormone.

a. Adjusted for log-transformed TSH, Hamilton Rating Scale for Anxiety scores, Hamilton Rating Scale for Depression scores and systolic blood pressure.

We further evaluated the association of autoimmune thyroiditis and suicide attempt in patients with anxious depression and psychotic depression. Our findings align with those observed in the overall sample, highlighting a consistent pattern across different forms of depression. Among patients with anxious depression, the prevalence of suicide attempt was markedly higher in the autoimmune thyroiditis with abnormal TSH group (50.2%, *n* = 131) compared with both the autoimmune thyroiditis only group (27.8%, *n* = 15) and the non-autoimmune thyroiditis group (23.8%, *n* = 138). A similar trend was noted in patients with psychotic depression, where the autoimmune thyroiditis with abnormal TSH group exhibited a significantly elevated prevalence of suicide attempt (77.8%, *n* = 49), surpassing the rates in the autoimmune thyroiditis only (14.3%, *n* = 1) and non-autoimmune thyroiditis (37.6%, *n* = 38) groups.

### Characteristics of patients with MDD and autoimmune thyroiditis with abnormal TSH with and without a history of suicide attempt

As we only found an elevated risk of suicide attempt among patients with autoimmune thyroiditis with abnormal TSH compared with patients with non-autoimmune thyroiditis, we decided to further investigate the potential correlates of suicide attempt in this particular subgroup. The clinical characteristics and biochemical parameters of the patients with MDD and autoimmune thyroiditis with abnormal TSH who did and did not have a history of suicide attempt are presented in Table 3. No differences in demographic information (age, gender, age at onset, duration of MDD, level of education and married status) were found between the two groups. Compared with those without a history of suicide attempt, patients with a history of suicide attempt exhibited worse depressive, anxiety and psychotic symptoms. Regarding thyroid function, serum levels of TPOAb, TgAb and TSH were significantly higher in patients with a history of suicide attempt, whereas no significant differences were found in FT3 and FT4 levels in patients with versus those without a history of suicide attempt. Notably, patients with a history of suicide attempt were found to have significantly higher fasting glucose, total cholesterol, LDL-C, total triglycerides, systolic and diastolic blood pressure, but significantly lower HDL-C, compared with those without a history of suicide attempt.

**Table 3:** Characteristics of patients with major depressive disorder and autoimmune thyroiditis with abnormal thyroid stimulating hormone, with and without a history of suicide attempt

Characteristic	Suicide attempt			*P*-value^b^
	Overall, *N* = 399^a^	Without suicide attempt, *n* = 247^a^	With suicide attempt, *n* = 152^a^	
Age, years	36 (13)	36 (13)	36 (13)	0.91
Duration, months	7.3 (5.1)	7.2 (5.1)	7.5 (5.1)	0.58
Age at onset, years	36 (12)	36 (12)	36 (12)	0.92
Gender				0.33
Male	125 (31%)	73 (30%)	52 (34%)	
Female	274 (69%)	174 (70%)	100 (66%)	
Education level				0.29
Below college	271 (68%)	163 (66%)	108 (71%)	
College or above	128 (32%)	84 (34%)	44 (29%)	
Married				0.22
Unmarried	107 (27%)	61 (25%)	46 (30%)	
Married	292 (73%)	186 (75%)	106 (70%)	
HRSD scores	31.50 (2.70)	30.71 (2.43)	32.80 (2.63)	<0.001
HRSA scores	22.1 (3.6)	20.8 (2.9)	24.3 (3.6)	<0.001
Anxious depression	261 (65%)	130 (53%)	131 (86%)	<0.001
PANSS scores	9.9 (5.3)	8.3 (3.2)	12.5 (6.8)	<0.001
Psychotic depression	63 (16%)	14 (5.7%)	49 (32%)	<0.001
TSH, uIU/L	7.26 (2.08)	6.62 (1.83)	8.31 (2.04)	
TgAb, IU/L	266 (411)	250 (418)	291 (399)	
TPOAb, IU/L	221 (262)	173 (215)	301 (309)	
FT3, pmol/L	4.93 (0.74)	4.91 (0.73)	4.95 (0.74)	
FT4, pmol/L	16.7 (3.0)	16.7 (3.0)	16.8 (3.0)	
Glucose, mmol/L	5.62 (0.66)	5.54 (0.61)	5.76 (0.72)	
Total cholesterol, mmol/L	5.78 (1.17)	5.54 (1.12)	6.17 (1.14)	
HDL-C, mmol/L	1.11 (0.31)	1.15 (0.33)	1.06 (0.28)	
LDL-C, mmol/L	3.27 (0.83)	3.16 (0.82)	3.45 (0.82)	
Total triglycerides, mmol/L	2.29 (1.01)	2.15 (0.95)	2.51 (1.05)	
BMI, kg/m^2^	24.44 (2.04)	24.45 (1.86)	24.42 (2.30)	0.92
SBP, mmHg	125 (9)	123 (8)	127 (10)	<0.001
DBP, mmHg	79 (7)	78 (6)	80 (7)	0.002
LnTSH	1.94 (0.28)	1.85 (0.26)	2.09 (0.26)	<0.001
LnFT3	1.58 (0.15)	1.58 (0.15)	1.59 (0.15)	0.65
LnFT4	2.80 (0.18)	2.80 (0.18)	2.80 (0.18)	0.71
LnTPOAb	4.75 (1.21)	4.54 (1.14)	5.08 (1.24)	<0.001
LnTgAb	4.83 (1.26)	4.73 (1.29)	5.00 (1.19)	0.035
LnGlucose	1.72 (0.12)	1.71 (0.11)	1.74 (0.13)	0.003
LnTC	1.73 (0.21)	1.69 (0.21)	1.80 (0.20)	<0.001
LnTG	0.74 (0.43)	0.68 (0.42)	0.83 (0.43)	<0.001
LnLDL-C	1.15 (0.28)	1.11 (0.29)	1.20 (0.27)	0.001
LnHDL-C	0.07 (0.29)	0.09 (0.30)	0.02 (0.26)	0.010

a. Mean (s.d.) or *n* (%).

b. Welch two sample *t*-test or Pearson's chi-squared test.

HRSD, Hamilton Rating Scale for Depression; HRSA, Hamilton Rating Scale for Anxiety; PANSS, Positive and Negative Syndrome Scale; TSH, thyroid stimulating hormone; TPOAb, thyroid peroxidase antibody; TgAb, antithyroglobulin; FT3, free triiodothyronine; FT4, free thyroxine; HDL-C, high-density lipoprotein; LDL-C, low-density lipoprotein; BMI, body mass index; SBP, systolic blood pressure; DBP, diastolic blood pressure; LnTSH, log-transformed TSH; LnFT3, log-transformed FT3; LnFT4, log-transformed FT4; LnTPOAb, log-transformed thyroid peroxidase antibody; LnTgAb, log-transformed antithyroglobulin; LnGlucose, log-transformed glucose; LnTC, log-transformed total cholesterol; LnTG, log-transformed total triglycerides; LnLDL-C, log-transformed LDL-C; LnHDL-C, log-transformed HDL-C.

### Independent risk factors for suicide attempt in patients with MDD and autoimmune thyroiditis with abnormal TSH

Finally, we assessed the independent risk factors for suicide attempt in patients with MDD and comorbid autoimmune thyroiditis with abnormal TSH (Table 4). We found that HRSA score (odds ratio 1.32, 95% CI 1.21–1.81; *P* < 0.001), lnTPOAb (odds ratio 1.44, 95% CI 1.17–1.76; *P* < 0.001) and lnTSH (odds ratio 4.56, 95% CI 1.67–12.45; *P* = 0.003) were independently associated with a higher risk of suicide attempt in patients with MDD and autoimmune thyroiditis and abnormal TSH.

**Table 4:** Logistic regression model for history of suicide attempt in patients with major depressive disorder and autoimmune thyroiditis with thyroid stimulating hormone

Characteristic	Odds ratio	95% CI	*P*-value
HRSA	1.32	1.21–1.81	<0.001
lnTSH	4.56	1.67–12.45	0.003
lnTPOAb	1.44	1.17–1.76	<0.001

The dependent variable was history of suicidal attempt, whereas HRSD scores, HRSA scores, PANSS scores, lnTSH, lnTPOAb, lnTgAb, lnGlucose, lnHDL-C, lnLDL-C, lnTG, SBP and DBP were the independent variables. HRSA, Hamilton Rating Scale for Anxiety; lnTSH, log-transformed thyroid stimulating hormone; lnTPOAb, log-transformed thyroid peroxidase antibody; HRSD, Hamilton Rating Scale for Depression; PANSS, Positive and Negative Syndrome Scale; lnTgAb, log-transformed antithyroglobulin; lnGlucose, log-transformed glucose; lnHDL-C, log-transformed HDL-C; lnLDL-C, log-transformed LDL-C; lnTG, log-transformed total triglycerides; SBP, systolic blood pressure; DBP, diastolic blood pressure.

## Discussion

To our knowledge, this study is the first to investigate the prevalence and clinical correlates of suicide attempt in patients with MDD and comorbid autoimmune thyroiditis. Our main findings included (a) approximately three in ten patients with FEDN MDD were comorbid with autoimmune thyroiditis; (b) patients with autoimmune thyroiditis and abnormal TSH levels were twice as likely to report suicide attempt compared with non-autoimmune thyroiditis groups, whereas no difference was noted in suicide attempt prevalence between non-autoimmune thyroiditis and autoimmune thyroiditis only groups; and (c) anxiety, TSH and TPOAb were independently associated with suicide attempt in patients with FEDN MDD and autoimmune thyroiditis with abnormal TSH.

Our study revealed a notably higher prevalence of autoimmune thyroiditis in patients with MDD compared with the general Chinese population.^18–20^ This aligns with a large-scale meta-analysis indicating a positive correlation between autoimmune thyroiditis and depression.^14^ To date, the relationship between autoimmune thyroiditis and depression presented considerable variability in findings across different samples. In line with our results, several studies in psychiatric out-patient settings have identified a higher susceptibility to MDD and anxiety symptoms in patients with autoimmune thyroiditis.^21,22^ Conversely, some community-based studies have not found a significant link between autoimmune thyroiditis and depressive symptoms.^23^ This discrepancy may be attributed to differences in depression severity between community and clinical samples, and the varied methodologies employed for assessment (self-reported questionnaires versus clinical interviews). Another possible explanation for the heightened prevalence of autoimmune thyroiditis in our study could be the distinctive nature of our sample, consisting of individuals with severe depression who had not yet received treatment for either their depressive symptoms or autoimmune thyroiditis.

Our study demonstrated, for the first time, that autoimmune thyroiditis subgroups exhibit differential clinical characteristics. Specifically, the autoimmune thyroiditis with abnormal TSH subgroup was associated with more severe clinical symptoms and an increased risk of suicide attempt, whereas no significant differences were observed between the autoimmune thyroiditis only and non-autoimmune thyroiditis groups in these respects. These findings underscore the potential significance of abnormal TSH levels, primarily indicative of subclinical hypothyroidism in our participants, in the relationship between autoimmune thyroiditis and clinical manifestations in MDD. Consistent with our findings, previous studies have documented the substantial association of subclinical hypothyroidism with worse clinical symptoms in MDD.^24–26^ Our results, if confirmed in further large-scale studies, highlight the importance of autoimmune thyroiditis severity levels in both clinical practice and future research.

Consistent with prior studies in patients with MDD,^10,12^ we found significantly higher autoimmune thyroid antibodies and TSH levels in patients with a history of suicide attempt. Furthermore, TSH and TPOAb levels were found to be independently related to history of suicide attempt in patients with autoimmune thyroiditis with abnormal TSH. There are several possible explanations for our findings. First, increased TPOAb levels, as a marker of autoimmune disease, might be associated with disturbances in the kynurenine pathway, monoamine metabolism, hypothalamic-pituitary-adrenal axis and inflammation, which may contribute to suicide attempt.^27^ Second, previous studies suggested that HPT axis dysfunction could be related to abnormalities in neurotransmitters (e.g. 5-hydroxytryptamine, dopamine and norepinephrine).^28,29^ Further studies are warranted to discover the potential biological mechanism linking TPOAb, TSH and suicide attempt in MDD.

In addition to TPOAb and TSH, the present study also found that severe anxiety was independently associated with suicide attempt in patients with autoimmune thyroiditis with abnormal TSH. Anxiety is the most common comorbidity of MDD, and is associated with severe depression, functional impairment and poor clinical outcomes.^30–32^ A large number of studies support the role of anxiety in suicide among patients with MDD. For example, Sanches et al found that the severity of anxiety was positively associated with suicidal thoughts among out-patients with MDD, and those with severe anxiety were 6.75 times more likely to report suicidal ideation.^33^ Wu et al demonstrated that both anxiety symptoms and anxiety disorders could increase the risk of suicide attempt in patients with MDD.^34^ Taken together, our findings emphasise the need for special attention to suicide attempt in patients with MDD and anxiety.

The present study has several limitations. First, as this study was cross-sectional, a causal relationship between suicide attempt and autoimmune thyroiditis in patients with MDD could not be established. Second, this study was conducted at a single hospital and included only Han Chinese patients with severe depressive symptoms. Further, several subtypes of MDD, such as atypical depression, were not measured in our study. Whether our findings can be applied to patients with other subtypes of MDD and individuals with mild-to-moderate depressive symptoms remains to be explored. Third, suicide attempt is highly associated with some sociocultural and biological factors, such as negative life events, inflammation and other autoimmune disorders, which were not analysed in this study. Future studies could benefit from including these measurements to provide a more comprehensive understanding of the association between autoimmune thyroiditis and suicide attempt in MDD. Fourth, suicide attempt was assessed with a single item, without an assessment of attempt lethality. Employing standardised and validated suicide assessments, such as the Sheehan Suicidality Tracking Scale and the Columbia Suicide Severity Rating Scale, might be more informative. Therefore, further multi-centre longitudinal studies are warranted to determine the causal relationship between autoimmune thyroiditis and suicide attempt, and to elucidate the underlying biological basis.

In conclusion, the present study shows that patients with MDD and autoimmune thyroiditis with abnormal TSH are at a higher risk for suicide attempt. TPOAb, TSH and anxiety are all independently associated with suicide attempt in this population, and regular thyroid tests are warranted.

## Supporting information

Luo et al. supplementary material Luo et al. supplementary material

## Data Availability

The data that support the findings of this study are available from the corresponding author, N.Y., upon reasonable request.
