# Supplementary material for: Prevalence and clinical correlates of suicide attempts in patients with first-episode drug-naïve major depressive disorder and comorbid autoimmune thyroiditis
Source: BJPsych Open. 2024 Apr 30;10(3):e95. doi: 10.1192/bjo.2024.48 (PMC11060091; doi:10.1192/bjo.2024.48)
Supplement: Luo et al. supplementary material [file S2056472424000486sup001.docx]

Supplementary material for “Prevalence and clinical correlates of suicide attempts in first-episode, never treated major depressive disorder patients with comorbid autoimmune thyroiditis”

Figure S1 Results of LASSO regression

Table S1 Correlation matrix of thyroid indexes and clinical symptoms


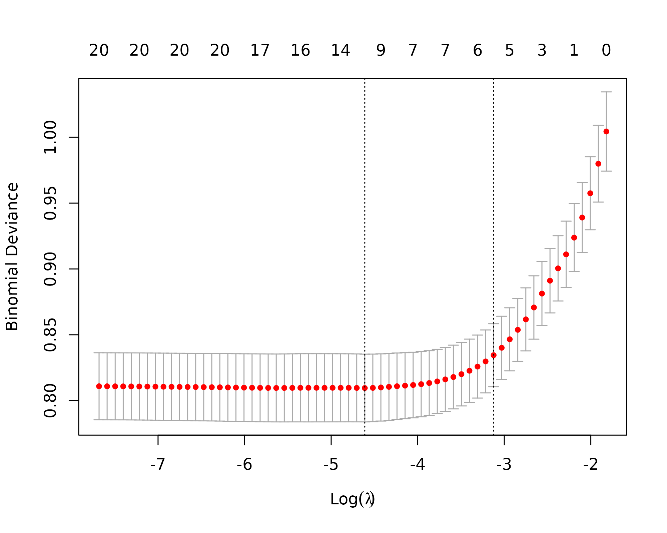

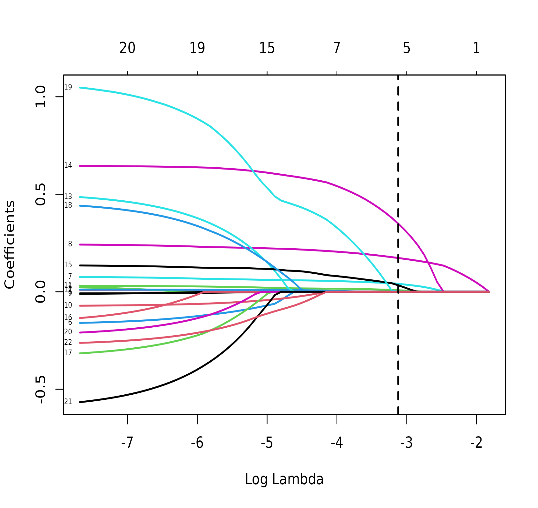


**Figure S1 Results of LASSO regression. (a) Lasso Regression Cross-Validation Plot; (b) Lasso Regression Coefficient Path Plot**

**Table S1 Correlation matrix of thyroid indexes and clinical symptoms**

|  | HAMD | HAMA | PANSS-P |
| --- | --- | --- | --- |
| lnTSH | 0.429^***^ | 0.234^***^ | 0.276^***^ |
| lnTgAb | 0.159^***^ | 0.200^***^ | 0.132^***^ |
| lnTPOAb | 0.189^***^ | 0.211^***^ | 0.154^***^ |
| lnFT3 | 0.033 | 0.022 | 0.002 |
| lnFT4 | 0.007 | 0.026 | 0.020 |

*** p<0.001

Note: HAMD = Hamilton Depression Rating Scale, HAMA = Hamilton Anxiety Rating Scale, PANSS-P = the Positive and Negative Syndrome Scale positive subscale, TSH = thyroid-stimulating hormone, FT3 = free triiodothyronine, FT4 = free thyroxine, TgAb = antithyroglobulin, TPOAb = thyroid peroxidases antibody.
